# Risk of Recurrent Noninfectious Uveitis After Coronavirus Disease 2019 Vaccination in the United States

**DOI:** 10.1016/j.xops.2024.100474

**Published:** 2024-01-20

**Authors:** Anika Kumar, D. Claire Miller, Yuwei Sun, Benjamin F. Arnold, Nisha R. Acharya

**Affiliations:** 1 F.I. Proctor Foundation, University of California, San Francisco, California; 2 Department of Ophthalmology, University of California, San Francisco, California; 3 Department of Epidemiology and Biostatistics, University of California, San Francisco, California; 4 Institute for Global Health Sciences, University of California, San Francisco, California

**Keywords:** COVID-19, COVID-19 vaccination, Incidence, Noninfectious uveitis, Recurrence

In a self-controlled case series analysis using a large United States claims database, an increased risk of noninfectious uveitis following COVID-19 vaccination was found in individuals with a prior history of the disease.

Prior studies have found that the risk of new onset noninfectious uveitis (NIU) is minimally or not increased after coronavirus disease 2019 (COVID-19) vaccination.^1^^,^^2^ However, questions remain regarding the risk of uveitis recurrence post-COVID-19 vaccination in those with a prior history of the disease.^3^ This study evaluated the association between COVID-19 vaccination and risk of recurrent NIU.

This study used deidentified claims data from Optum Labs Data Warehouse (Optum Labs), which includes medical and pharmacy claims and enrollment information from > 200 million commercial and Medicare Advantage enrollees in the United States.^4^ The study population consisted of patients with 1 to 4 doses of a COVID-19 vaccine (BNT162b2, mRNA-1273, or Ad26.COV2.S) from December 11, 2020 to November 30, 2021. Patients ≥ 5 years of age who were continuously enrolled with medical and pharmacy coverage from 730 days preceding their first vaccine record (index date) with a NIU diagnosis prior to the study period were included. Patients with an International Classification of Diseases, 10th Revision code for infectious uveitis in the lookback period or during the observation period were excluded.

Noninfectious uveitis outcome events were identified as encounters with an ophthalmologist/optometrist with an International Classification of Diseases, 10th Revision code for NIU in the first or second diagnosis code position and a prescription for a new or escalated corticosteroid treatment within 7 days of diagnosis or an administration of a local steroid injection or implant. Codes used to identify vaccines, NIU, infectious uveitis, and corticosteroids were based on previous NIU studies conducted from this database.^1^

Using a self-controlled case series design, risk of NIU recurrence was compared in exposed postvaccine risk periods and unexposed control periods within each individual. Each individual’s study period spanned from their index date to 1/31/2022 or date of disenrollment from the medical plan, if earlier, and was divided into control and risk periods. Risk periods were defined as the 60 days after each vaccination and were censored early if another COVID-19 vaccine was administered or if the study period ended within the 60 days. To account for the healthy vaccinee bias, a 30-day window prior to a COVID-19 booster dose administration was excluded from the analysis. All other time during the study period was considered to be unexposed control period (Fig S1, available at www.ophthalmologyscience.org). Only individuals with a NIU outcome in the study period contributed to the risk estimation.

Conditional Poisson regression with an offset of the natural log of the length of the entire interval was used to estimate incidence rate ratios (IRRs), 95% confidence intervals, and *P* values for the risk of NIU recurrence. Time since last NIU flare was adjusted for by dividing the observation period into 30-day subintervals based on days since last flare; terms for these subintervals were included in the regression model. Subgroup analyses were conducted by age group, vaccine type, use of systemic corticosteroid, or other systemic immunosuppressive therapy in the 90 days prior to first vaccination and anatomic location of NIU. Statistical analyses were performed in R (version 4.1.0, R Foundation for Statistical Computing). Two-tailed statistical tests were used, and *P* < 0.05 was considered statistically significant. This study adhered to the tenets of the Declaration of Helsinki and was granted a waiver of consent by the Institutional Review Board of the University of California San Francisco because the data were deidentified.

A total of 412 individuals experienced an NIU outcome event in the study period and were included in the analysis (mean age [standard deviation] = 51.5 [15.1] years, 60.7% female, Table S1, available at www.ophthalmologyscience.org). The median amount of time since last NIU flare to the first vaccination was 303 days (interquartile range = 98.8–710 days).

In the postvaccine risk intervals, which cumulatively spanned 111 person-years throughout the study period, 209 NIU outcomes were identified. In the unexposed control intervals, which spanned 184 person-years cumulatively, 203 NIU outcomes were identified. After adjusting for time since last NIU flare, the IRR comparing NIU risk in the postvaccine risk intervals to the unexposed intervals was 1.29 (95% confidence interval: 1.02–1.62, *P* = 0.03) (Fig 2). Figure 2 also presents the IRRs for the subgroup analyses. In a subgroup analysis by baseline immunomodulatory therapy use, risk of NIU recurrence was higher in patients with exposure to these medications, although not statistically significant. In a subgroup analysis by anatomic subtype of NIU, an increased risk of recurrence was identified in the intermediate/posterior/panuveitis subgroup (IRR = 1.67, 95% confidence interval: 1.04–2.68, *P* = 0.03).

**Figure 2:**
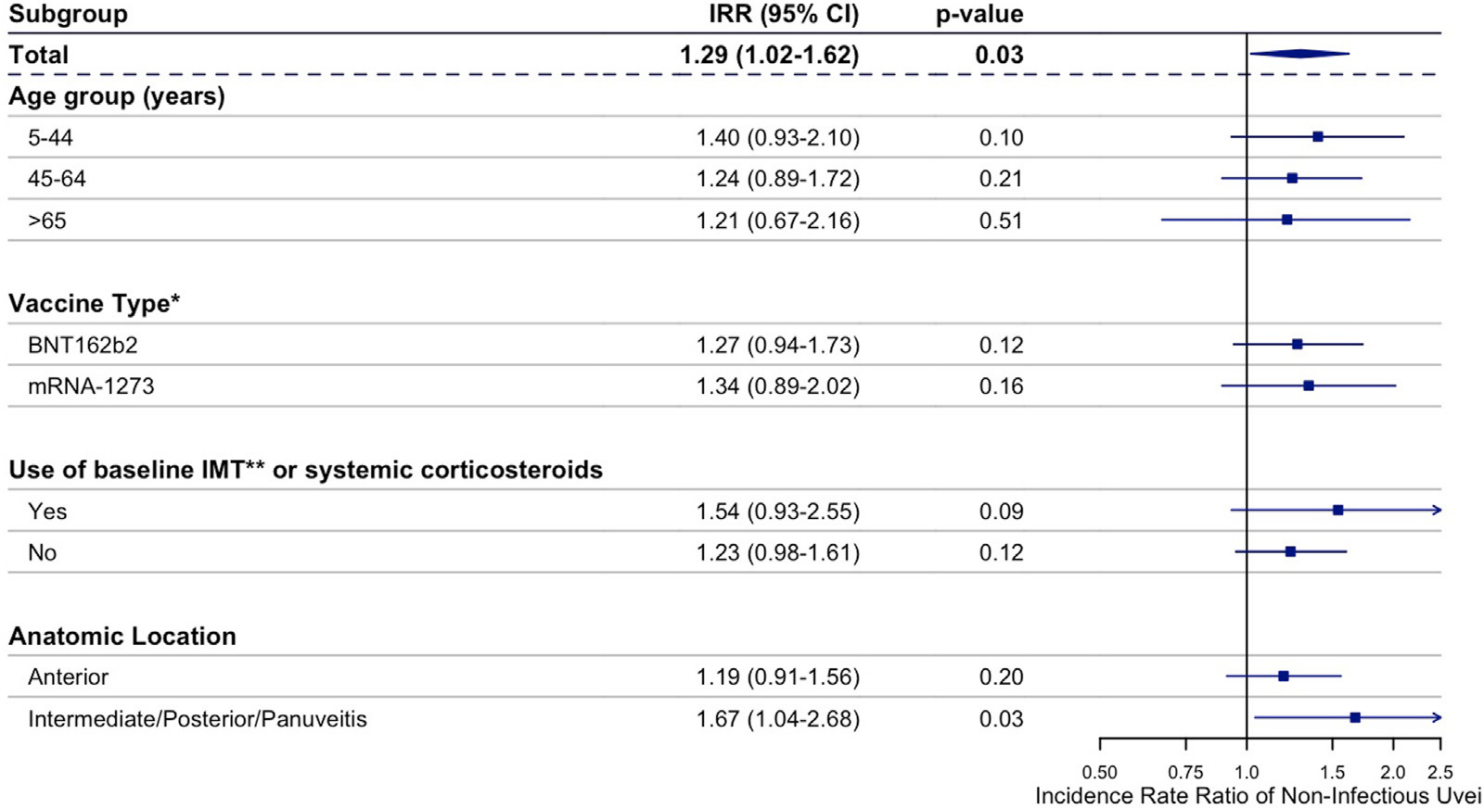
Incidence rate ratios of recurrent noninfectious uveitis in subgroups from the self-controlled case series analysis. ∗Vaccine subgroups only include patients who only received 1 vaccine type. Patients who received multiple vaccine types were excluded from the subgroup analysis, but not the overall analysis. Sample size for Ad26.COV2.S subgroup was insufficient to obtain incidence rate ratio and profile confidence interval (CI). ∗∗Immunomodulatory therapy (IMT) included methotrexate, azathioprine, mycophenalate mofetil, mycophenolic acid, cyclosporine, leflunomide, tacrolimus, infliximab, adalimumab, certolizumab pegol, golimumab, sarilumab, tocilizumab, siltuximab. rituximab, abatacept, ocrelizumab, secukinumab, ustekinumab, anakinra, canakinumab, daclizumab, and interferon beta. IRR = incidence rate ratio.

This retrospective self-controlled case series analysis from a large United States population found a mildly increased risk of NIU after COVID-19 vaccination in patients with a prior history of the disease. Patients with intermediate, posterior, or panuveitis had the most pronounced risk of recurrence. These findings suggest a potential benefit to heightened monitoring in the postvaccination period in patients with a history of NIU. Proposed theories for the molecular mechanism of vaccine-associated NIU include vaccine peptide fragments inducing self-antibodies through molecular mimicry, vaccine adjuvants triggering the innate immune response, and type 3 delayed hypersensitivity reactions to vaccine antigens.^5^ Individuals with a prior history of NIU may be predisposed to this type of immune dysregulation.

Prior studies point to no increased or marginally increased risk of new onset NIU after COVID-19 vaccination, but less is known about risk in populations with a history of NIU.^1^ An Israeli-based study that evaluated risk of NIU after COVID-19 vaccination found a possible increased risk of recurrence in the subgroup of patients with a prior history of NIU compared with historical controls, prompting the need for further study.^2^ Additionally, an observational New Zealand-based study found a rate of 20.7 uveitis flares per person-month after vaccination (compared to 12.4 per person-month at baseline) but was not able to statistically compare the rates or account for preexisting risk factors.^6^ The current study focusing on patients with a history of NIU provides robust estimates of the risk of recurrence post-COVID-19 vaccination and utilizes a self-controlled case series design to minimize bias.

One limitation of our study is that small sample sizes in some subgroups precluded identification of those most likely to experience increased postvaccine NIU risk. Another limitation with claims data is the possibility of NIU outcome misclassification. For example, it is possible that patients with new or escalated steroid prescriptions were receiving them for something other than a NIU event and may have been incorrectly classified as an event. Additionally, claims data do not provide information on whether immunomodulatory medications were stopped temporarily after COVID-19 vaccinations, which may have introduced bias toward finding a signal. However, the American College of Rheumatology guidelines published during the study period only recommended withholding medications for patients on mycophenolate, methotrexate, janus kinase inhibitors, abatacept, and rituximab for 1 to 4 weeks after vaccination; no modifications were recommended for patients on other immunosuppressive therapies.^7^ Finally, though time since last NIU flare was adjusted for, there may have been residual confounding from this variable given limited understanding of how recurrence risk changes over time.

This study found an increased risk of NIU after COVID-19 vaccination in those with a history of the disease. Further study is warranted and may have clinical implications for this population, including the need for postvaccination monitoring.

## Supplementary Data

Supplementary Figure 1 Illustration of self-controlled case series study design in a single patient. COVID-19 = coronavirus diease 2019.
