# Supplementary material for: Risk of Recurrent Noninfectious Uveitis After Coronavirus Disease 2019 Vaccination in the United States
Source: Ophthalmol Sci. 2024 Jan 20;4(4):100474. doi: 10.1016/j.xops.2024.100474 (PMC11141252; doi:10.1016/j.xops.2024.100474)
Supplement: Supplementary Figure 1 — Illustration of self-controlled case series study design in a single patient. COVID-19 = coronavirus diease 2019. [file mmc1.pdf]

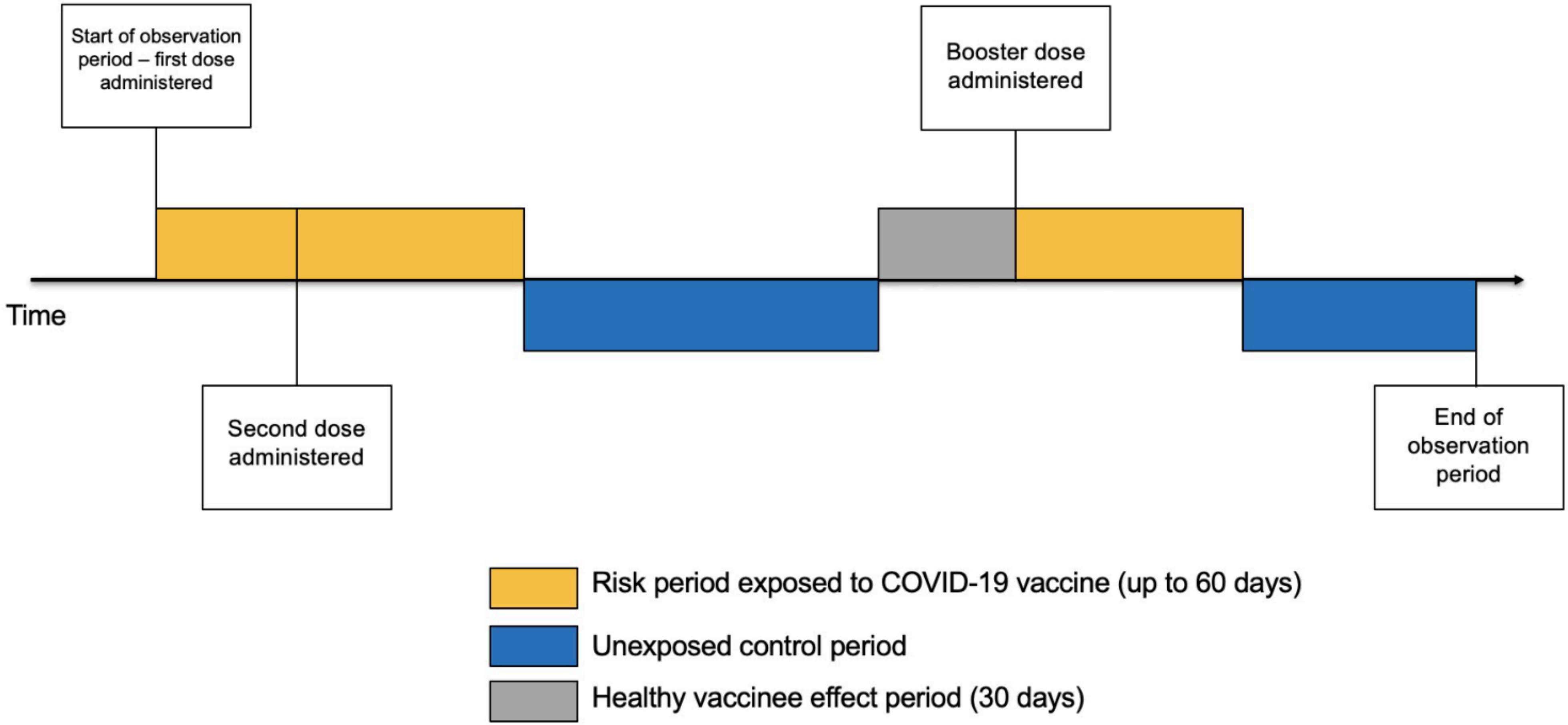
Start of observation period – first dose administered
Booster dose administered
Time
Second dose administered
End of observation period
Risk period exposed to COVID-19 vaccine (up to 60 days)
Unexposed control period
Healthy vaccinee effect period (30 days)
