# Supplementary material for: Risk of Recurrent Noninfectious Uveitis After Coronavirus Disease 2019 Vaccination in the United States
Source: Ophthalmol Sci. 2024 Jan 20;4(4):100474. doi: 10.1016/j.xops.2024.100474 (PMC11141252; doi:10.1016/j.xops.2024.100474)
Supplement: Supplementary Table 1 [file mmc2.pdf]

Table 1: Characteristics of study population and COVID-19 vaccine doses included in the self-controlled case series analysis

Patient-level Characteristics	N patients (%) (N=412)[a]
**Age**	
Mean years (SD)	51.5 (15.1)
Median years [Q1, Q3]	53.0 [40.8, 63.0]
**Gender**	
Female	250 (60.7%)
Male	162 (39.3%)
**Race[b]**	
Asian	31 (7.5%)
Black	68 (16.5%)
Hispanic	34 (8.3%)
White	267 (64.8%)
Unknown	12 (2.9%)
**Insurance type**	
Commercial	366 (88.8%)
Medicare Advantage	46 (11.2%)
**History of Autoimmune disease**	148 (35.9%)
**Charlson Comorbidity Index**	
Mean (SD)	1.95 (2.18)
Median [Q1, Q3]	1.00 [0, 3.00]
**Time since last NIU flare (days)**	
Mean (SD)	358 (270)
Median [Q1, Q3]	303 [98.8, 710]
**Visits with eye care provider for NIU in previous 2 years**	
Mean (SD)	5.91 (4.93)
Median [Q1, Q3]	5.00 [2.00, 8.00]
**Received treatment in previous 90 days**	
Systemic steroid, ocular steroid injection/implant, or IMT	128 (31.1%)
Topical ophthalmic steroids	164 (39.8%)
**Uveitis anatomic location**	
Anterior	>288 (>69.9%)
Intermediate/posterior/panuveitis	113 (27.4%)
Unknown	<11 (<2.7%)
**Dose-level Characteristics**	**N doses (%) (N=862)**
**Type of vaccination**	
BNT162b2	512 (59.4%)
mRNA-1273	324 (37.6%)
Ad26.COV2.S	26 (3.0%)
**Dose administered**	
1st	412 (47.8%)
2nd	314 (36.4%)
3rd or 4th	136 (15.8%)
**Site of vaccination**	
Mass immunization center	14 (1.6%)
Office	278 (32.3%)

Outpatient hospital	84 (9.7%)
Pharmacy	444 (51.5%)
Other	42 (4.9%)
**Month of vaccination**	
Dec 2020 through Feb 2021	132 (15.3%)
Mar 2021 through May 2021	436 (50.6%)
June 2021 through Aug 2021	88 (10.3%)
Sep 2021 through Dec 2021	206 (23.9%)

Abbreviations: NIU = non-infectious uveitis; IMT = immunomodulatory therapy

[a] Small cell sizes are reported as <11 to protect patient privacy.

[b] Ethnicity is assigned by an external vendor who uses a rule-based system that combines analysis of first names, middle names, surnames, and surname prefixes and suffixes with geographic criteria. Optum Labs then assigns these ethnicity values into one of five compliance-determined race/ethnicity code values: W (Non-Hispanic White), B (Non-Hispanic Black), H (Hispanic), A (Asian), and U (Unknown).
